# Supplementary material for: A foundational large language model for edible plant genomes
Source: Commun Biol. 2024 Jul 9;7:835. doi: 10.1038/s42003-024-06465-2 (PMC11233511; doi:10.1038/s42003-024-06465-2)
Supplement: Supplementary file 2 — Supplementary Information [file 42003_2024_6465_MOESM2_ESM.pdf]

Supplementary Information

Supplementary Figures

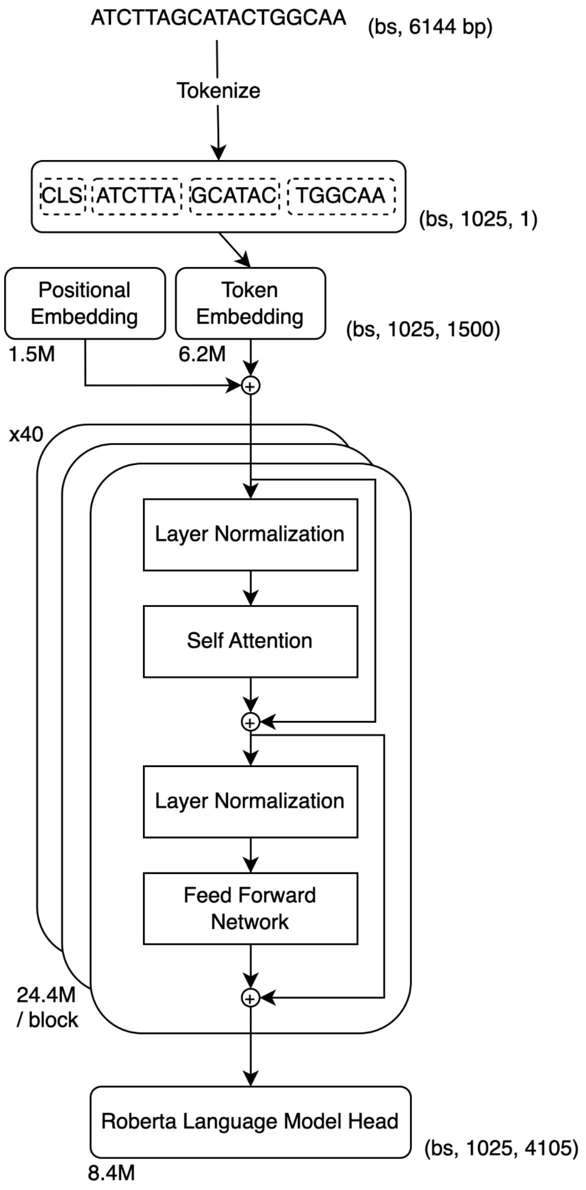

Supplementary Figure 1. A schematic representation of the AgroNT model architecture. Output shapes are shown as tuples on the right side of the blocks. The number of trainable parameters for different parts of the model are shown on the left side of the blocks. The acronym ”bs” indicates batch size.

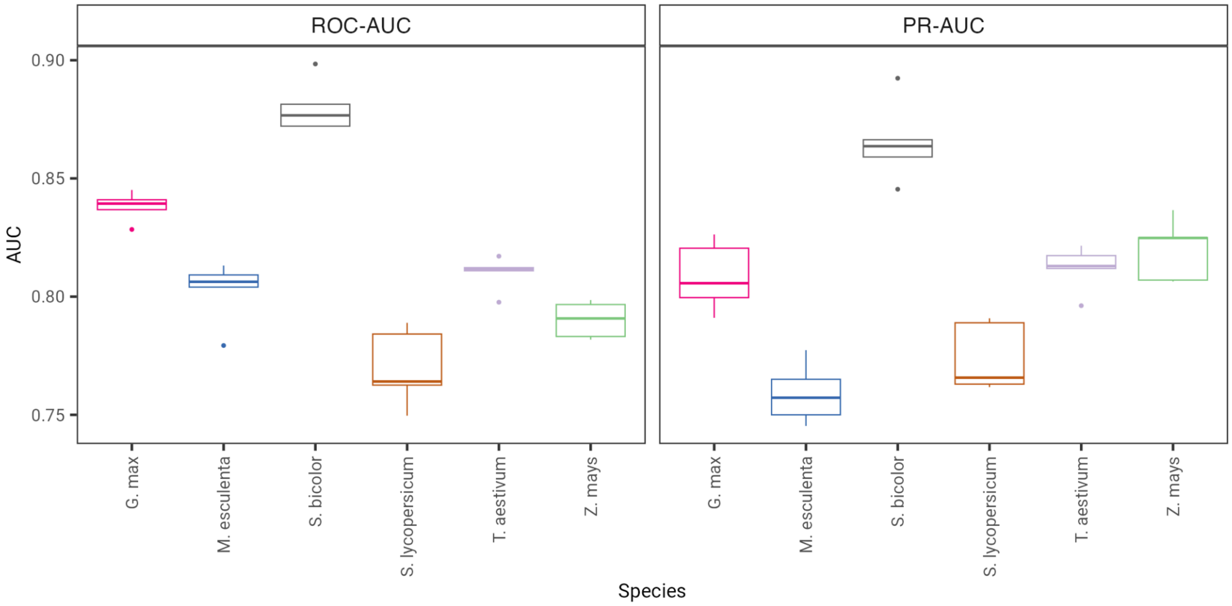

Supplementary Figure 2. Receiver operating characteristic area under the curve (ROC-AUC) and precision-recall area under the curve (PR-AUC) for classifying long non-coding RNAs and mRNA sequences across six species. The boxplots show the AUCs for 5 independently resampled matched numbers of mRNAs used as negative sequences.

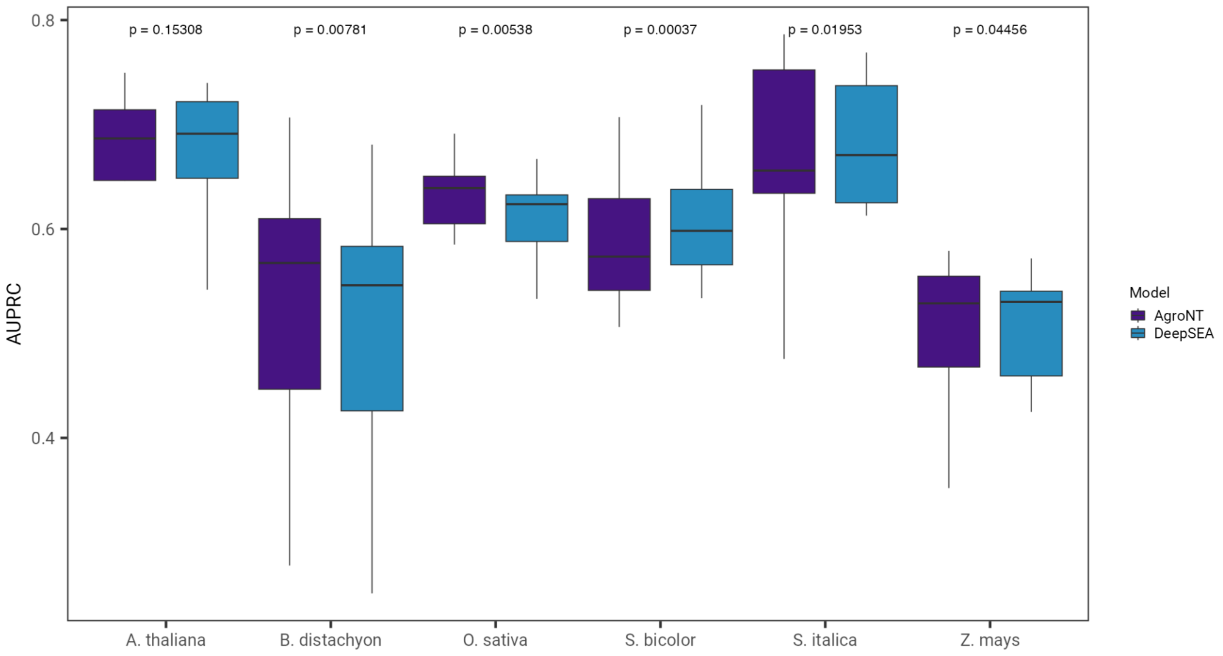

Supplementary Figure 3. The performance of AgroNT in predicting chromatin profiles across species and tissues is measured using the area under the curve of the precision-recall curve (AUPRC). P-values shown are based on a two-sided Wilcoxon signed test comparing the performance of AgroNT with a model based on the DeepSEA architecture.

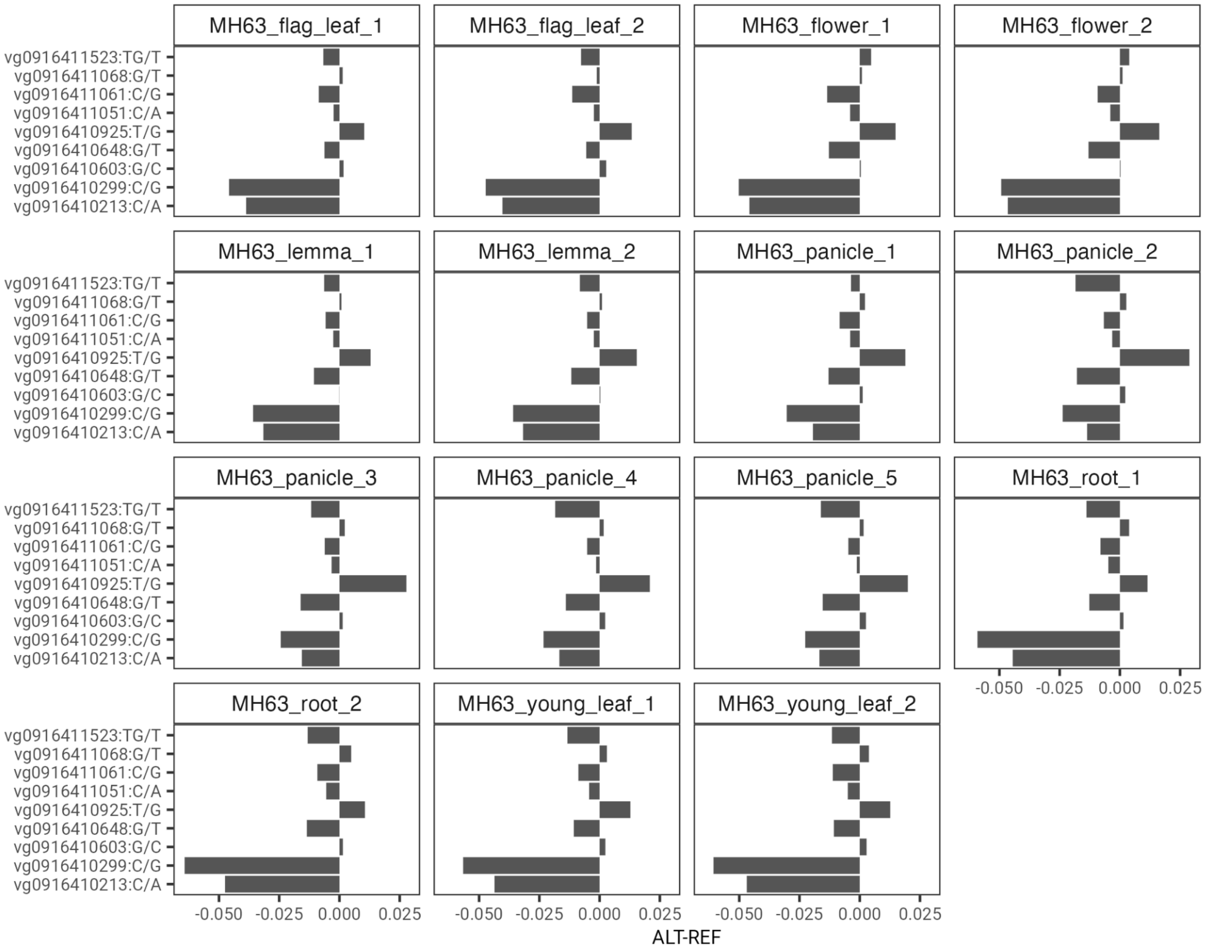

Supplementary Figure 4. The predicted effects of nine non-coding variants of the *DEP1* gene, previously associated with leaf variation and crop yield in *Oryza sativa*. Barplots show the difference in the predicted effects of alternative and reference alleles on chromatin accessibility across multiple tissues. Each variant is identified by its identification label, followed by the reference and alternative alleles.

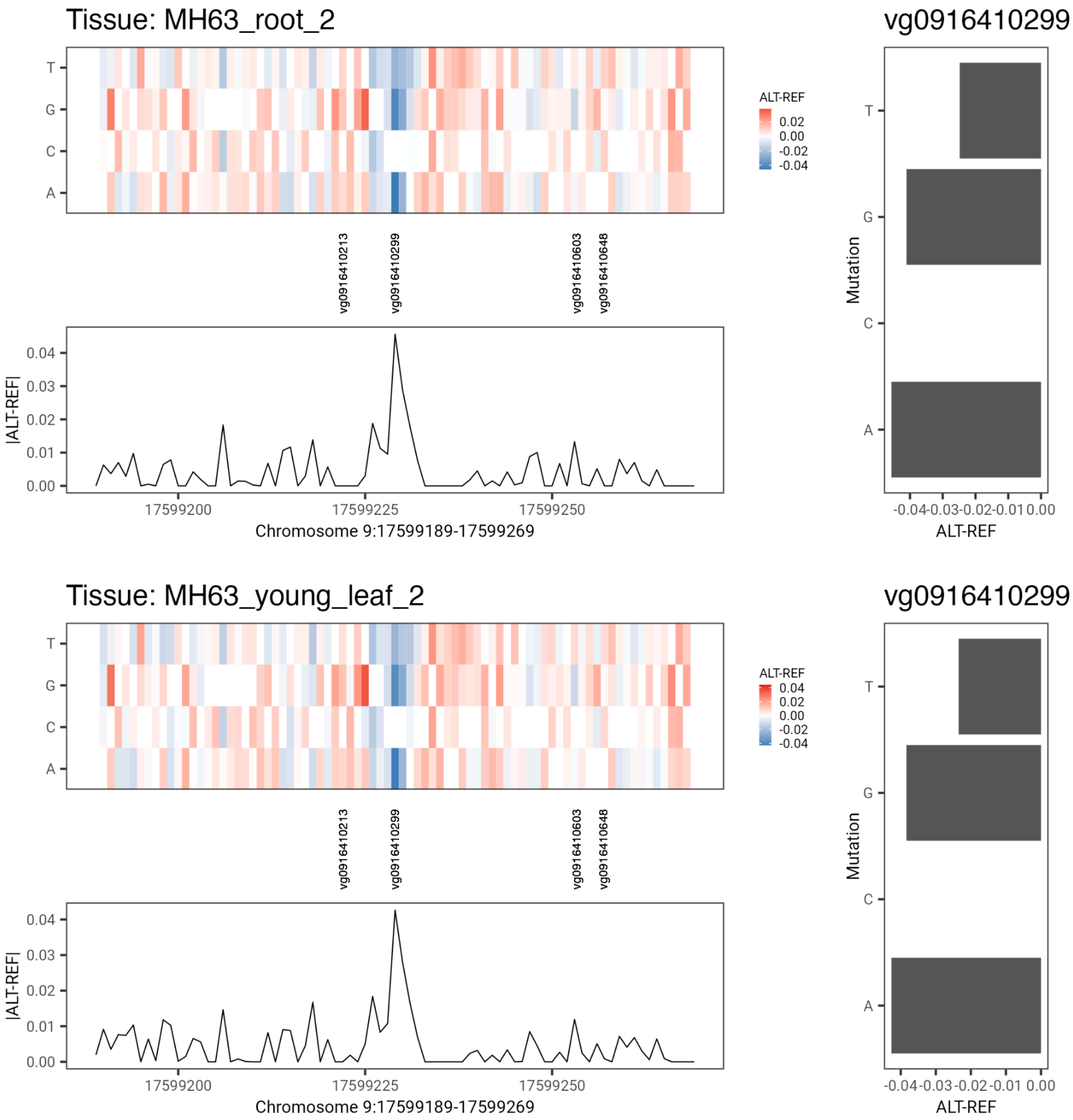

Supplementary Figure 5. *In silico* saturation mutagenesis analysis across a region overlapping the vg0916410299 variant. The resulting *in silico* saturation mutagenesis map illustrates the effect of mutating every base of a given sequence, quantified as the difference between the predicted effects of alternative and reference alleles on chromatin accessibility in root (upper plot) and leaf (bottom plot). The upper panel of each plot displays the mutagenesis analysis, the middle panel highlights the position of the vg0916410299, along with nearby variants, and the bottom panel shows the absolute value of the difference between the predicted effects of the strongest alternative and reference allele. The bar plot on the right side of each plot shows the effects of the 3 mutations tested for the vg0916410299 variant. Note that the reference allele for vg0916410299 is ‘C’, and as such, it shows a predicted effect of 0.

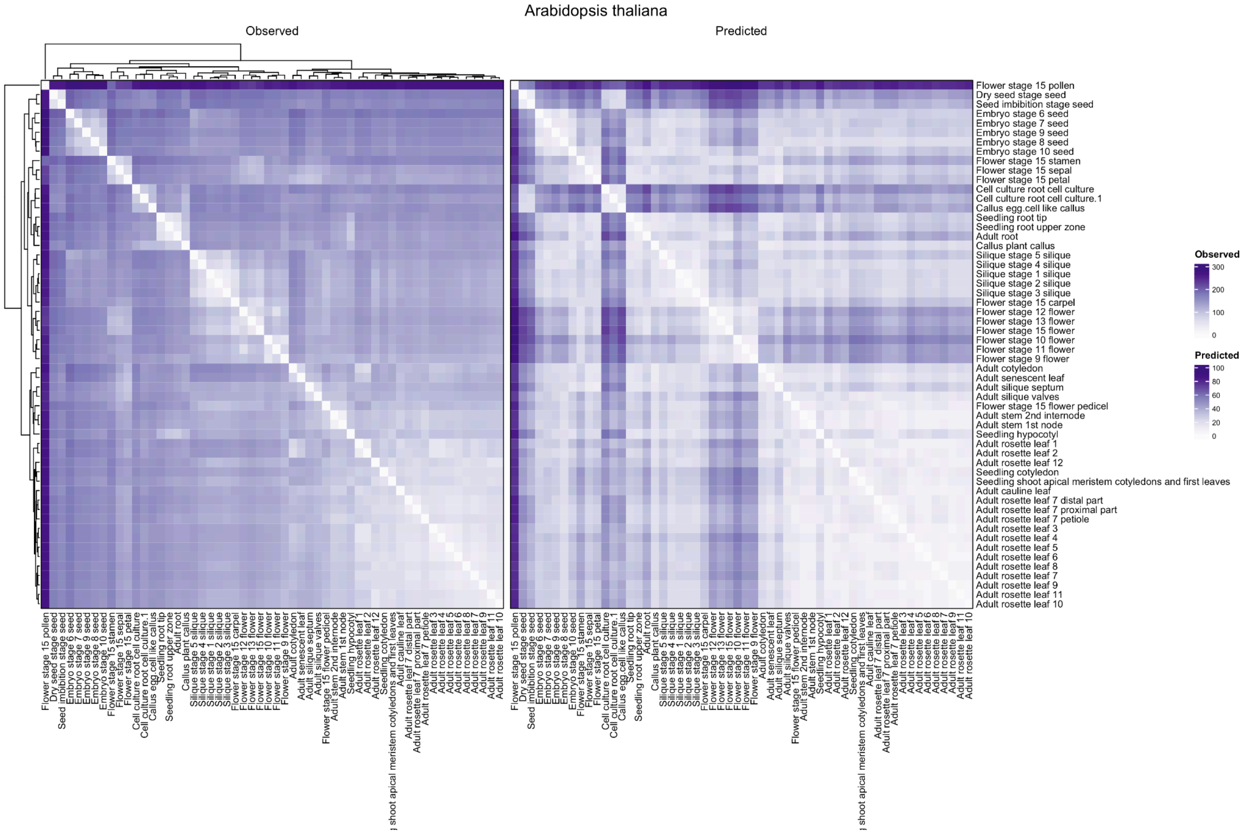

Supplementary Figure 6. Tissue expression profiles between observed and predicted gene expression levels on holdout genes for *Arabidopsis thaliana*. The order of the cells in the heatmap is based on a hierarchical clustering using the euclidean distance of the observed gene expression levels across tissues.

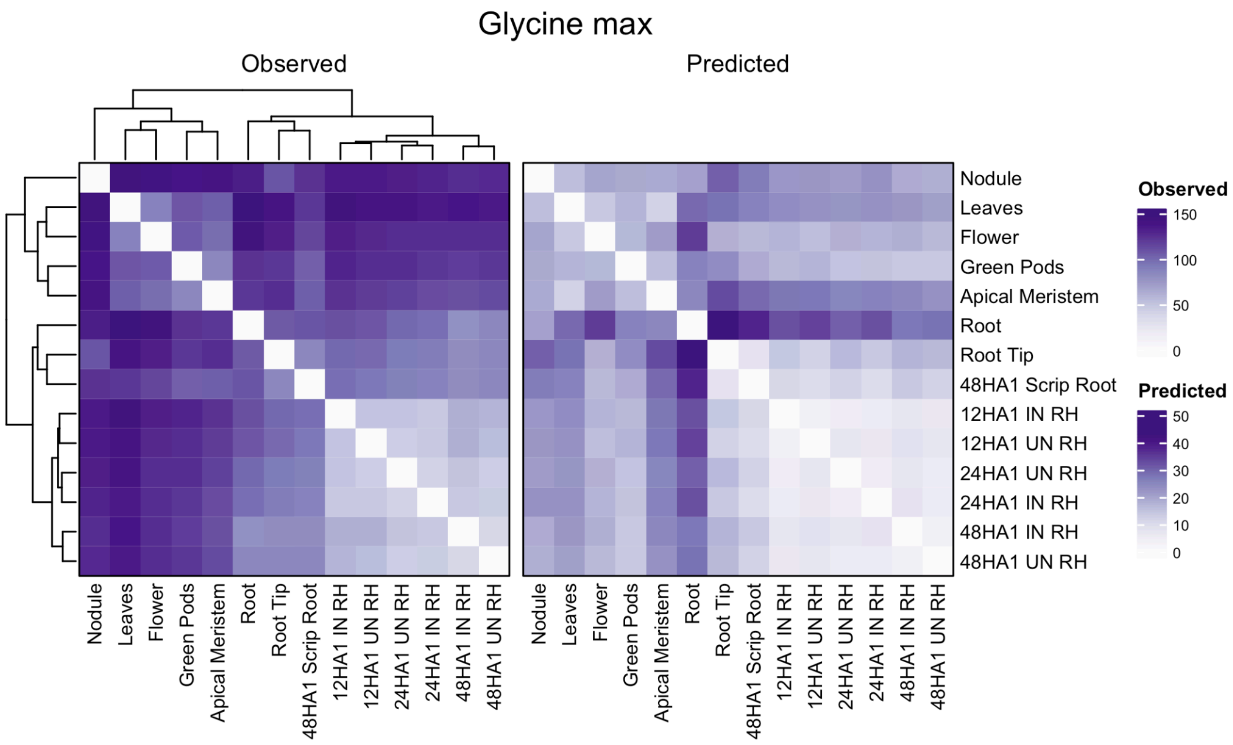

Supplementary Figure 7. Tissue expression profiles between observed and predicted gene expression levels on holdout genes for *Glycine max*. The order of the cells in the heatmap is based on a hierarchical clustering using the euclidean distance of the observed gene expression levels across tissues.

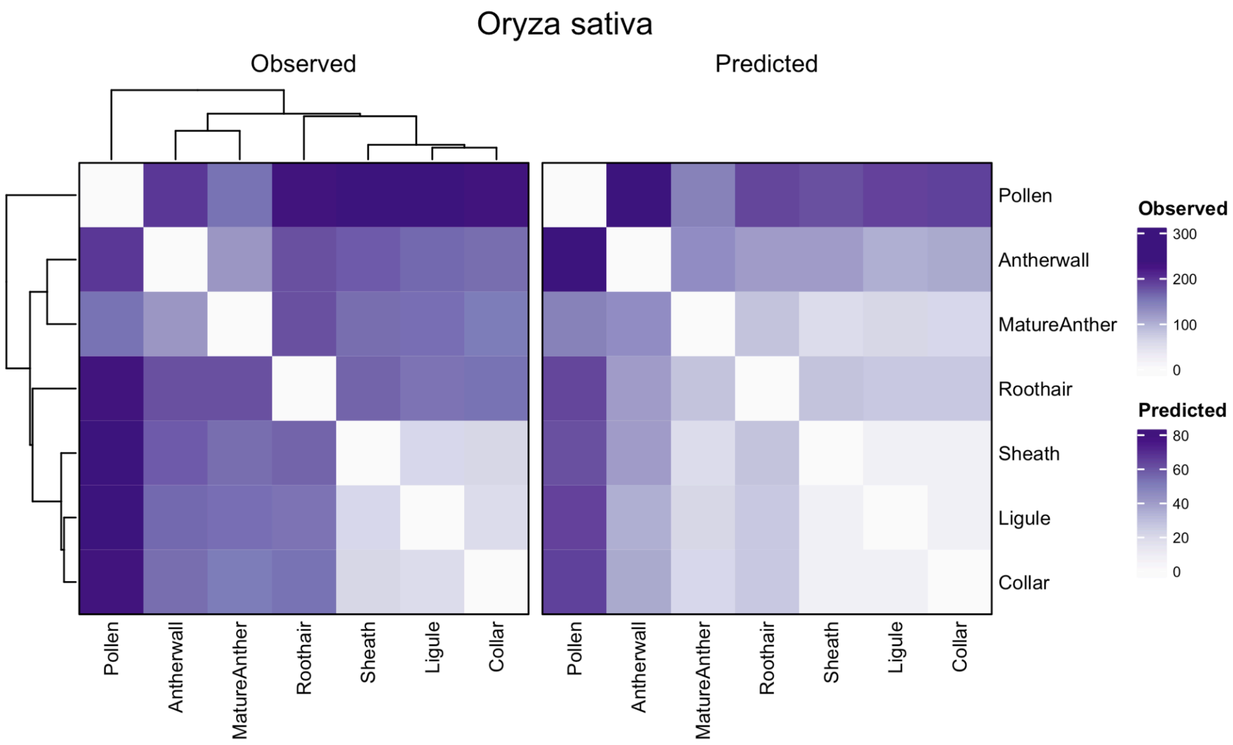

Supplementary Figure 8. Tissue expression profiles between observed and predicted gene expression levels on holdout genes for *Oryza sativa*. The order of the cells in the heatmap is based on a hierarchical clustering using the euclidean distance of the observed gene expression levels across tissues.

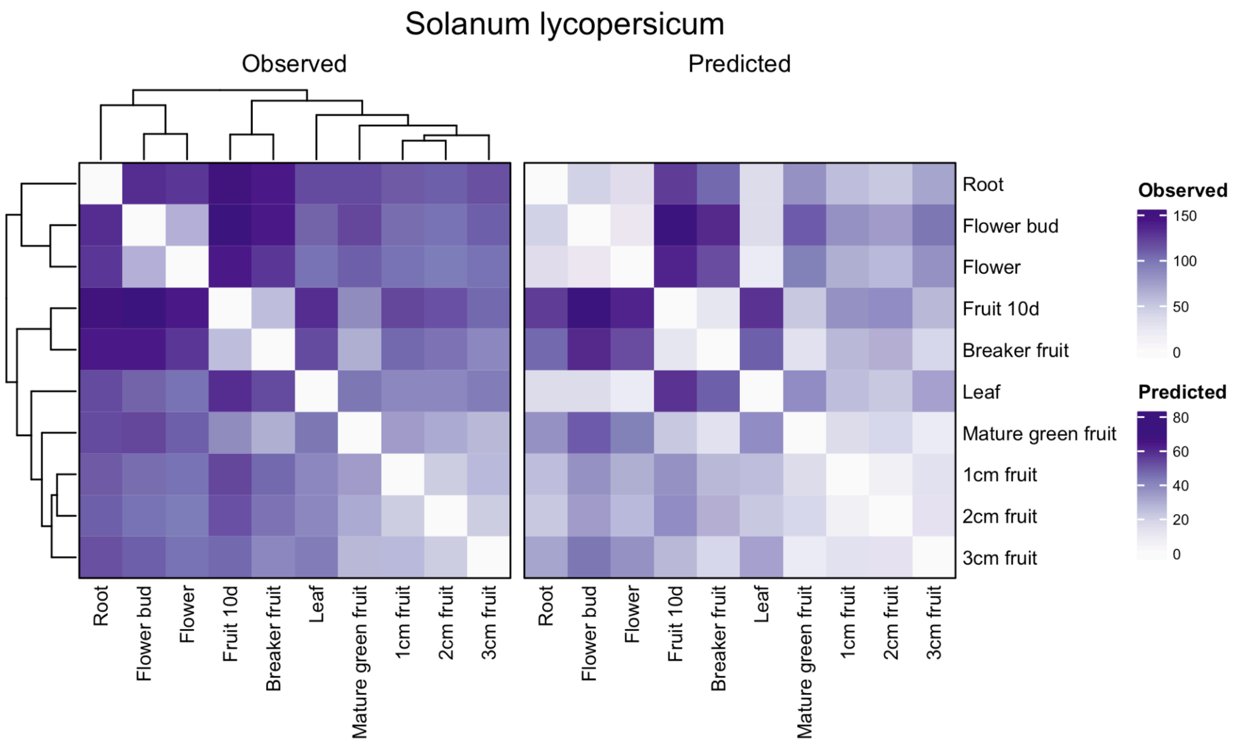

Supplementary Figure 9. Tissue expression profiles between observed and predicted gene expression levels on holdout genes for *Solanum lycopersicum*. The order of the cells in the heatmap is based on a hierarchical clustering using the euclidean distance of the observed gene expression levels across tissues.

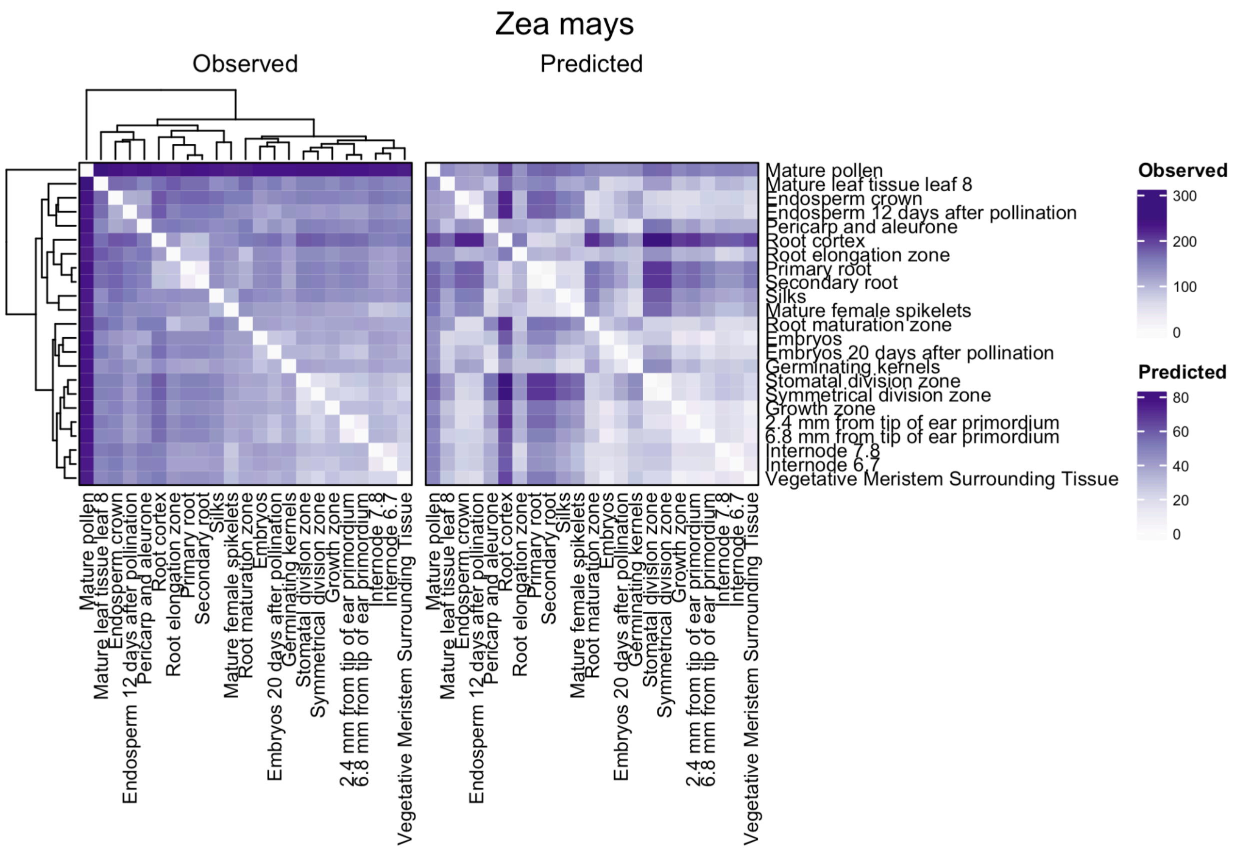

Supplementary Figure 10. Tissue expression profiles between observed and predicted gene expression levels on holdout genes for *Zea mays*. The order of the cells in the heatmap is based on a hierarchical clustering using the euclidean distance of the observed gene expression levels across tissues.

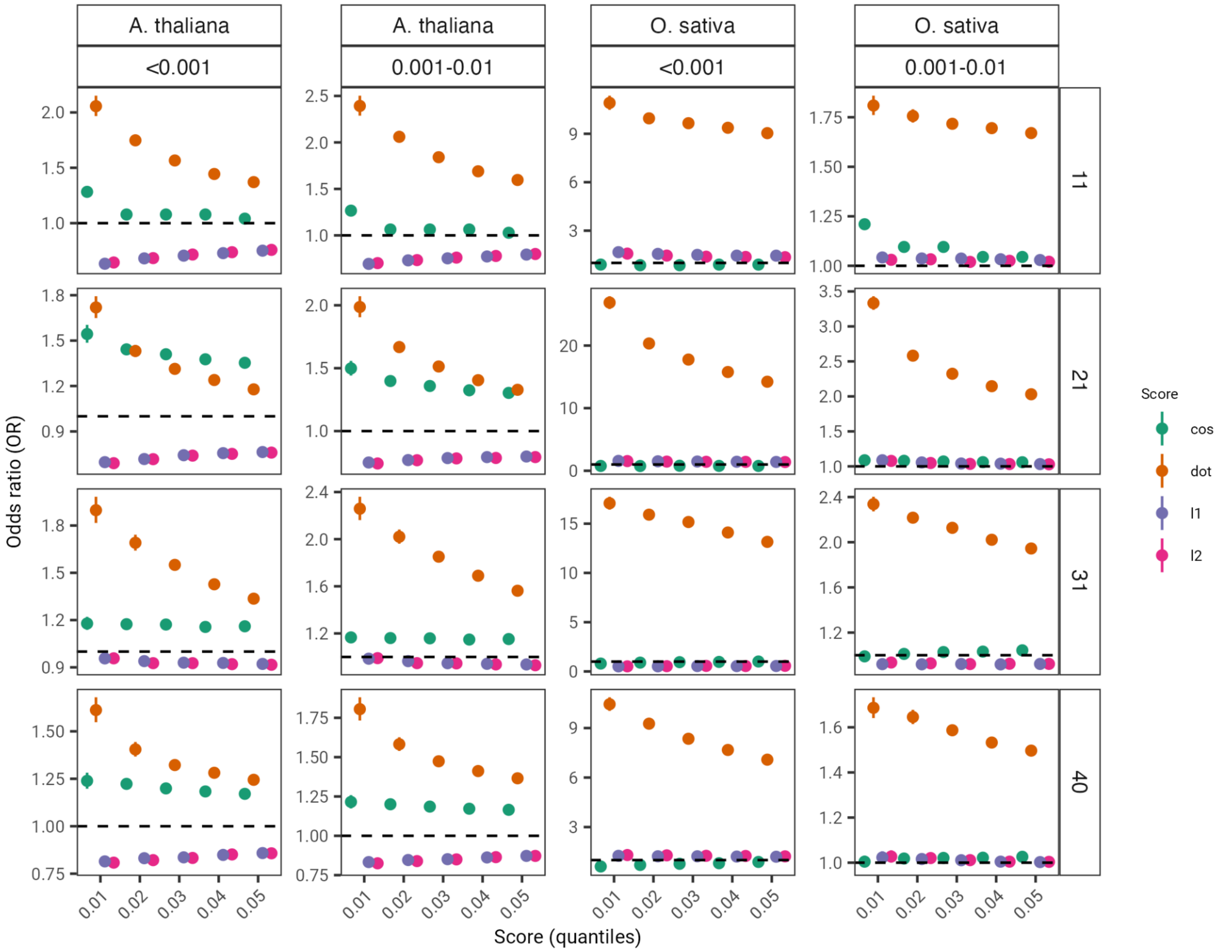

Supplementary Figure 11. Odd ratios (OR) across different score thresholds used to assess enrichment of two frequency classes of variants: lower than 0.1% and between 0.1% and 1%. The performance of four zeroshot scores are shown for *Arabidopsis thaliana* and *Oryza sativa*. Error bars denote 95% confidence intervals. Dashed black line indicates an OR of 1.

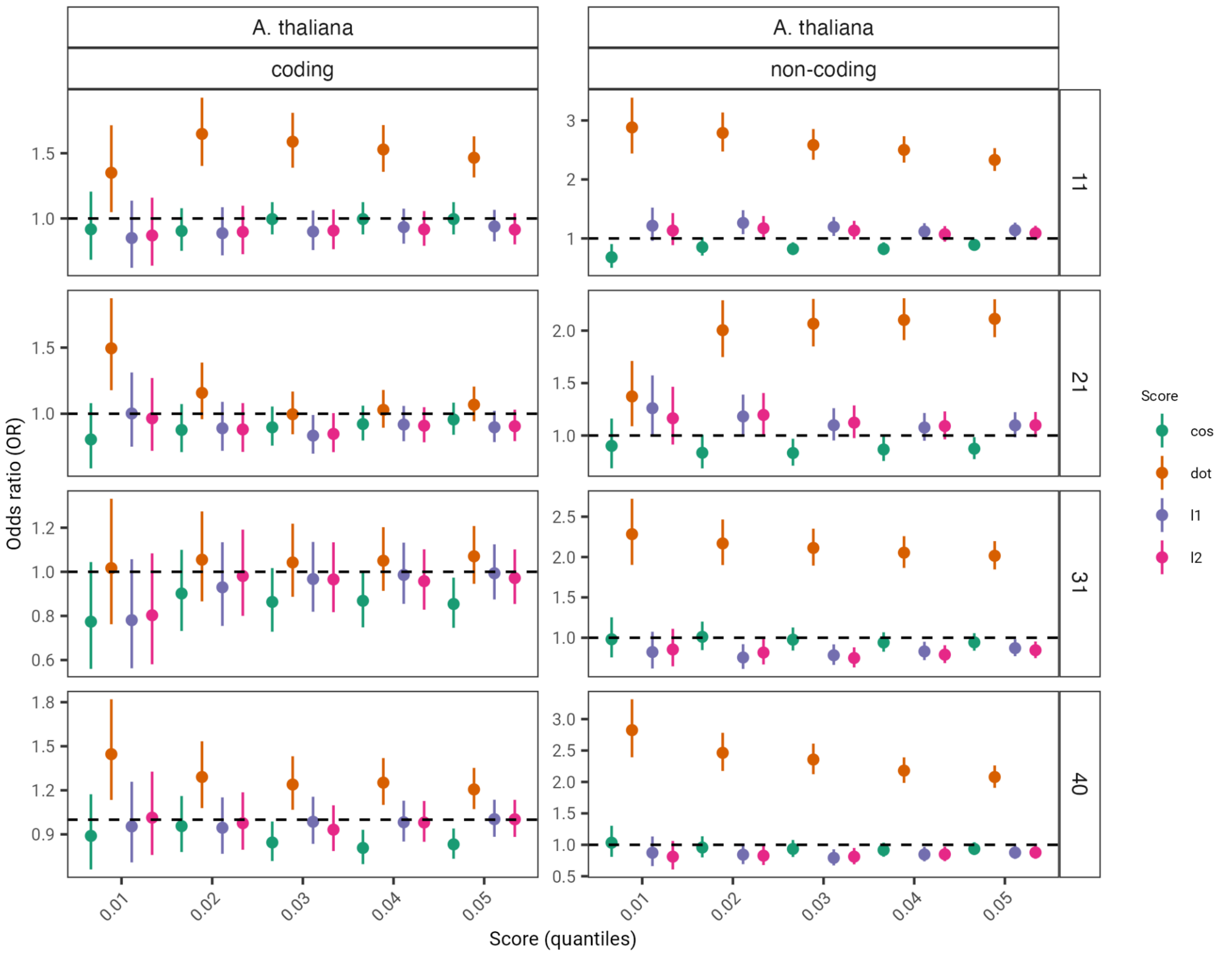

Supplementary Figure 12. Odd ratios (OR) across different score thresholds used to assess enrichment of coding and non-coding GWAS variants for *Arabidopsis thaliana*. The performance of four zero-shot scores are shown. Error bars denote 95% confidence intervals. Dashed black line indicates an OR of 1.

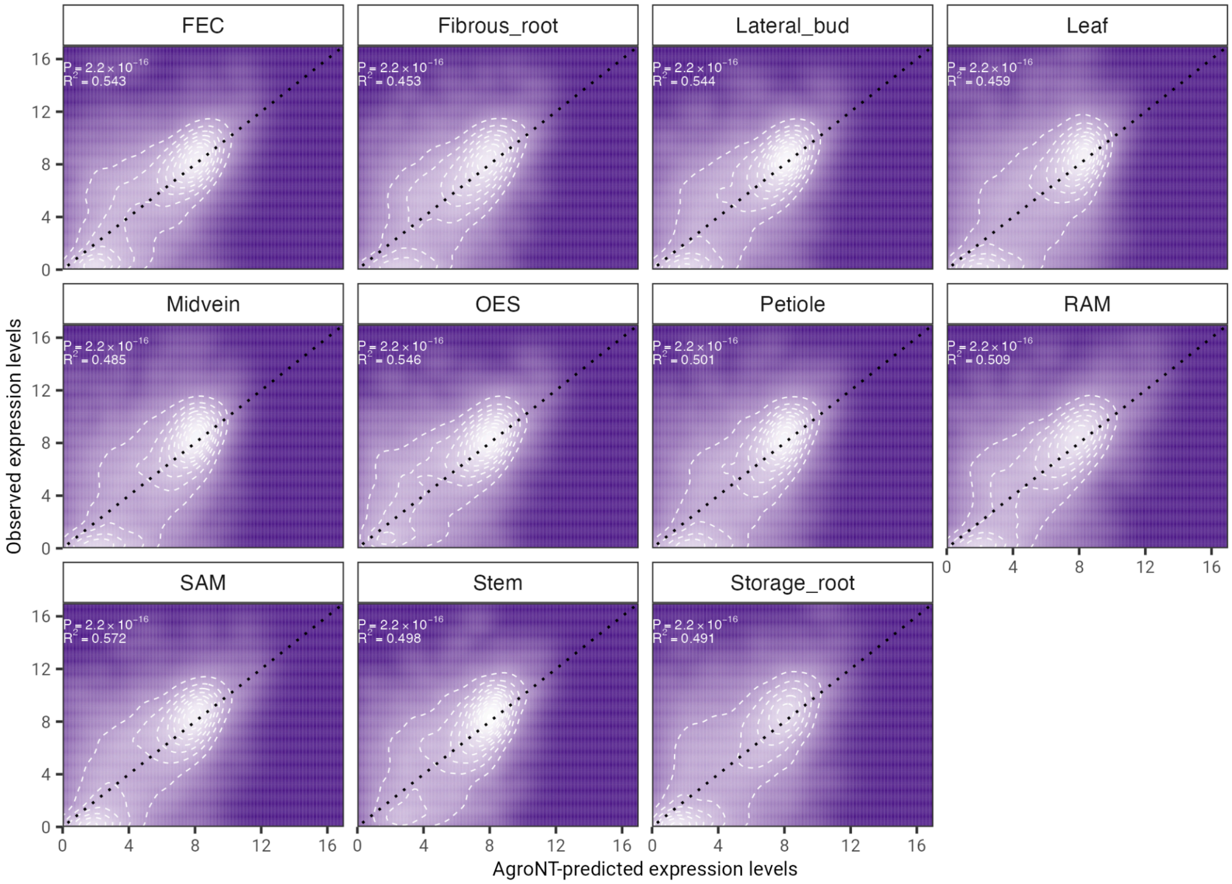

Supplementary Figure 13. Gene expression prediction on holdout genes across cassava tissues. The coefficient of determination (R2) from a linear model and associated P-values between predicted and observed values are shown.

Supplementary Tables

**Supplementary Table 1. Model architecture and training hyperparameters of the Agronomic Nucleotide Transformer.**	
**Tokenizer**	
Alphabet type	k mers
Alphabet size	4105
K mer	6
Prepend class token	true
Append end of sequence token	false
Max positions in tokens (not including class token)	1024
**Architecture**	
Token dropout	false
Positional embedding type	learned
Bias token embeddings	false
Embedding size	1500
Key size	75
Add bias to keys/values	true
# Attention heads	20
Feed forward network embedding size	5120
Add bias to feed forward network	true
Feed forward network activation function	gelu
# Attention layers	40
Language model head type	roberta
Total # of parameters	991,260,405
**Training**	
Masking ratio	0.15
Masking probability	0.8
Warmup initial learning rate	5.00E-05
Warmup end learning rate	1.00E-04
Number of warmup updates	64,000
Total number of update steps	315,000
Optimizer	Adam
Optimizer beta1	0.9
Optimizer beta2	0.999
Batch size (tokens)	1,500,000

Supplementary Table 2. R-squared values between observed and predicted expression across tissues on holdout genes for Arabidopsis thaliana.			
**Tissue**	**No. observations**	**R-squared**	**P-value**
adult_cauline_leaf_ERR3333391	3328	0.627	2.20E-16
adult_cotyledon_ERR3333412	3328	0.571	2.20E-16
adult_root_ERR3333396	3328	0.644	2.20E-16
adult_rosette_leaf_10_ERR3333423	3328	0.65	2.20E-16
adult_rosette_leaf_11_ERR3333424	3328	0.651	2.20E-16
adult_rosette_leaf_12_ERR3333425	3328	0.659	2.20E-16
adult_rosette_leaf_1_ERR3333413	3328	0.616	2.20E-16
adult_rosette_leaf_2_ERR3333414	3328	0.612	2.20E-16
adult_rosette_leaf_3_ERR3333415	3328	0.63	2.20E-16
adult_rosette_leaf_4_ERR3333416	3328	0.628	2.20E-16
adult_rosette_leaf_5_ERR3333417	3328	0.63	2.20E-16
adult_rosette_leaf_6_ERR3333418	3328	0.639	2.20E-16
adult_rosette_leaf_7_ERR3333419	3328	0.639	2.20E-16
adult_rosette_leaf_7_distal_part_ERR3333392	3328	0.628	2.20E-16
adult_rosette_leaf_7_petiole_ERR3333394	3328	0.635	2.20E-16
adult_rosette_leaf_7_proximal_part_ERR3333393	3328	0.633	2.20E-16
adult_rosette_leaf_8_ERR3333420	3328	0.648	2.20E-16
adult_rosette_leaf_9_ERR3333422	3328	0.638	2.20E-16
adult_senescent_leaf_ERR3333395	3328	0.585	2.20E-16
adult_silique_septum_ERR3333400	3328	0.609	2.20E-16
adult_silique_valves_ERR3333401	3328	0.619	2.20E-16
adult_stem_1st_node_ERR3333389	3328	0.637	2.20E-16
adult_stem_2nd_internode_ERR3333390	3328	0.632	2.20E-16
callus_egg.cell_like_callus_ERR3333402	3328	0.641	2.20E-16
callus_plant_callus_ERR3333403	3328	0.645	2.20E-16
cell_culture_root_cell_culture_ERR3333404	3328	0.683	2.20E-16
cell_culture_root_cell_culture_ERR3333405	3328	0.643	2.20E-16
dry_seed_stage_seed_ERR3333441	3328	0.575	2.20E-16
embryo_stage_10_seed_ERR3333440	3328	0.596	2.20E-16
embryo_stage_6_seed_ERR3333436	3328	0.547	2.20E-16
embryo_stage_7_seed_ERR3333437	3328	0.585	2.20E-16
embryo_stage_8_seed_ERR3333438	3328	0.603	2.20E-16
embryo_stage_9_seed_ERR3333439	3328	0.573	2.20E-16

flower_stage_10_flower_ERR3333427	3328	0.659	2.20E-16
flower_stage_11_flower_ERR3333428	3328	0.66	2.20E-16
flower_stage_12_flower_ERR3333429	3328	0.641	2.20E-16
flower_stage_13_flower_ERR3333430	3328	0.633	2.20E-16
flower_stage_15_carpel_ERR3333421	3328	0.647	2.20E-16
flower_stage_15_flower_ERR3333398	3328	0.634	2.20E-16
flower_stage_15_flower_pedicel_ERR3333397	3328	0.633	2.20E-16
flower_stage_15_petal_ERR3333399	3328	0.591	2.20E-16
flower_stage_15_pollen_ERR3333443	3328	0.346	2.20E-16
flower_stage_15_sepal_ERR3333388	3328	0.583	2.20E-16
flower_stage_15_stamen_ERR3333410	3328	0.545	2.20E-16
flower_stage_9_flower_ERR3333426	3328	0.683	2.20E-16
seed_imbibition_stage_seed_ERR3333442	3328	0.58	2.20E-16
seedling_cotyledon_ERR3333406	3328	0.636	2.20E-16
seedling_hypocotyl_ERR3333407	3328	0.658	2.20E-16
seedling_root_tip_ERR3333408	3328	0.604	2.20E-16
seedling_root_upper_zone_ERR3333409	3328	0.635	2.20E-16
seedling_shoot_apical_meristem_cotyledons_and_first_leaves_ERR3333411	3328	0.664	2.20E-16
silique_stage_1_silique_ERR3333431	3328	0.639	2.20E-16
silique_stage_2_silique_ERR3333433	3328	0.633	2.20E-16
silique_stage_3_silique_ERR3333432	3328	0.625	2.20E-16
silique_stage_4_silique_ERR3333434	3328	0.602	2.20E-16
silique_stage_5_silique_ERR3333435	3328	0.598	2.20E-16

**Supplementary Table 3. R-squared values between observed and predicted expression across tissues on holdout genes for Glycine max.**			
**Tissue**	**No. observations**	**R-squared**	**P-value**
Apical_Meristem_Stacey	4480	0.562	2.20E-16
Flower_Stacey	4480	0.495	2.20E-16
Green_Pods_Stacey	4480	0.535	2.20E-16
Leaves_Stacey	4480	0.482	2.20E-16
Nodule_Stacey	4480	0.478	2.20E-16
Root_Stacey	4480	0.592	2.20E-16
Root_Tip_Stacey	4480	0.527	2.20E-16
12HA1_IN_RH	4480	0.521	2.20E-16
12HA1_UN_RH	4480	0.526	2.20E-16
24HA1_IN_RH	4480	0.551	2.20E-16
24HA1_UN_RH	4480	0.535	2.20E-16
48HA1_IN_RH	4480	0.569	2.20E-16
48HA1_Scrip_Root	4480	0.526	2.20E-16
48HA1_UN_RH	4480	0.56	2.20E-16

**Supplementary Table 4. R-squared values between observed and predicted expression across tissues on holdout genes for Oryza sativa.**			
**Tissue**	**No. observations**	**R-squared**	**P-value**
Antherwall	3584	0.397	2.20E-16
Collar	3584	0.474	2.20E-16
Ligule	3584	0.453	2.20E-16
MatureAnther	3584	0.449	2.20E-16
Pollen	3584	0.304	2.20E-16
Roothair	3584	0.41	2.20E-16
Sheath	3584	0.448	2.20E-16

**Supplementary Table 5. R-squared values between observed and predicted expression across tissues on holdout genes for Solanum lycopersicum.**			
**Tissue**	**No. observations**	**R-squared**	**P-value**
1cm_fruit	3200	0.583	2.20E-16
2cm_fruit	3200	0.582	2.20E-16
3cm_fruit	3200	0.569	2.20E-16
breaker_fruit	3200	0.532	2.20E-16
flower	3200	0.531	2.20E-16
flower_bud	3200	0.532	2.20E-16
fruit_10d	3200	0.531	2.20E-16
leaf	3200	0.539	2.20E-16
mature_green_fruit	3200	0.554	2.20E-16
root	3200	0.55	2.20E-16

Supplementary Table 6. R-squared values between observed and predicted expression across tissues on holdout genes for Zea mays.

Tissue	No. observations	R-squared	P-value
Internode_6.7	4480	0.671	2.20E-16
Internode_7.8	4480	0.677	2.20E-16
Mature_pollen	4480	0.406	2.20E-16
Primary_root	4480	0.625	2.20E-16
Root_cortex	4480	0.578	2.20E-16
Root_elongation_zone	4480	0.614	2.20E-16
Root_maturation_zone	4480	0.646	2.20E-16
Secondary_root	4480	0.629	2.20E-16
Vegetative_Meristem_Surrounding_Tissue	4480	0.67	2.20E-16
X2.4_mm_from_tip_of_ear_primordium	4480	0.668	2.20E-16
X6.8_mm_from_tip_of_ear_primordium	4480	0.664	2.20E-16
embryos	4480	0.625	2.20E-16
embryos_20_days_after_pollination	4480	0.651	2.20E-16
endosperm_12_days_after_pollination	4480	0.645	2.20E-16
endosperm_crown	4480	0.593	2.20E-16
germinating_kernels	4480	0.619	2.20E-16
growth_zone	4480	0.639	2.20E-16
mature_female_spikelets	4480	0.66	2.20E-16
mature_leaf_tissue_leaf_8	4480	0.57	2.20E-16
pericarp_and_aleurone	4480	0.586	2.20E-16
silks	4480	0.61	2.20E-16
stomatal_division_zone	4480	0.649	2.20E-16
symmetrical_division_zone	4480	0.659	2.20E-16
