# Supplementary material for: A foundational large language model for edible plant genomes
Source: Commun Biol. 2024 Jul 9;7:835. doi: 10.1038/s42003-024-06465-2 (PMC11233511; doi:10.1038/s42003-024-06465-2)
Supplement: Supplementary file 3 — Description of Additional Supplementary Files [file 42003_2024_6465_MOESM3_ESM.pdf]

Description of Additional Supplementary Files

File name: Supplementary Data 1.
Description: Genome, annotation version, and source of reference genome pre-training.

File name: Supplementary Data 2.
Description: RNA sequencing dataset used for gene expression prediction.

File name: Supplementary Data 3.
Description: Genome, annotation version, and source of reference genomes used for gene expression prediction.
